# Too Much Salt to My Taste: An Entity to Think about in Neonatal Hypernatremia: A Case Report and Review of the Literature

**DOI:** 10.1155/2024/8838362

**Published:** 2024-03-08

**Authors:** Marwa El Masri, Lidiya Samotiy-Hanna, Ramy Ghabril, Yolla Nassif, Dany Al Hamod

**Affiliations:** ^1^Department of Pediatrics, Saint George University Medical Center, University of Balamand, Beirut, Lebanon; ^2^Department of Pediatric Nephrology, Saint George University Medical Center, University of Balamand, Beirut, Lebanon; ^3^Department of Pediatric and Neonatal Intensive Care, Saint George University Medical Center, University of Balamand, Beirut, Lebanon

## Abstract

In exclusively breastfed newborns, hypernatremic dehydration is associated with a free water deficit secondary to insufficient fluid intake. Failure of newborns to regain their birth weight by the 10th day of life should be investigated urgently. In this report, we present a case of a 2 -week-old girl who presented to our institution for 30% weight loss and was found to have severe hypernatremic dehydration associated with acute renal failure (creatinine 4 mg/dL). Upon further investigation, the breast milk sodium content was found to be extremely elevated (90 mEq/L). To our knowledge, the following reported case of severe neonatal hypernatremic dehydration associated with acute renal failure has the most elevated breast milk sodium content, serum sodium, and serum creatinine levels described in the literature. Thus, hypernatremic dehydration secondary to elevated breast milk content should always be borne in mind and investigated whenever suspected.

## 1. Introduction

Neonatal hypernatremia is a serious condition that is associated with increased morbidity and mortality [1]. Following normal postnatal diuresis, neonates are expected to lose 7% of their birth weight, which they typically regain by the 10th day of life [2]. Thus, weight loss of more than 7% poses serious concerns and needs to be urgently investigated.

Hypernatremia is associated with decreased fluid intake, excessive fluid loss, or excessive sodium intake [3]. Term healthy neonates who are discharged from the hospital at 48−72 h of life sometimes present back by the caregivers with concerns of dehydration, mostly secondary to difficulty feeding and expressing milk, leading to hypernatremic dehydration and requiring readmission [4]. In March 1962, an error in making proprietary milk led to mass accidental salt poisoning in infants with a fatality rate of 43% of the exposed newborns (6 out of 14) [5]. Therefore, an unusually high sodium content of breast milk should also be kept in mind as an etiology of hypernatremic dehydration in neonates.

Herein, we present the case of a 2 -week-old baby girl that presented to our institution with severe hypernatremic dehydration, associated with 30% weight loss and acute renal failure, whose mother turned out to have an extremely elevated sodium breast milk content of 90 mEq/L.

## 2. Case Report

A primigravida mother gave birth to a healthy girl at term via spontaneous vaginal delivery. The baby weighed 4080 g at birth and had no perinatal complications. At her 2 -week well-child check, she was found to have lost approximately 30% of her birth weight and weighed 2800 g. Her parents reported that she was exclusively breastfed every 2 hours for approximately 10 minutes on each breast and had an average of 6 wet diapers per day. The baby was active at home with no signs of lethargy. Pregnancy was uneventful, and the mother had two normal morphologic ultrasounds with no reported history of oligohydramnios or polyhydramnios.

On examination, the baby was alert and active but with dry mucous membranes, cracked lips, and capillary refill more than 3 seconds. The skin was soft and doughy. Laboratory studies revealed the following: sodium 196 mEq/L; potassium 6.7 mEq/L; chloride 155 mEq/L; bicarbonate 15 mEq/L; urea 440 mg/dL; creatinine 4.08 mg/dL; phosphorus 7.8 mg/dL, and uric acid 19.9 mg/dL. A urine dipstick showed a specific gravity of 1.019, 2+ protein, and 2+ glucose. Urinary sodium 152 mEq/L, urinary potassium 25.4 mEq/L, urinary creatinine 34.1 mg/dL, urine osmolarity 736 mosmol/kg, and serum osmolarity 475 mosmol/kg. Liver enzymes and coagulation studies were normal. Renal and bladder ultrasound was normal. Electrocardiogram was normal.

The baby was subsequently admitted for parenteral rehydration and correction of her hypernatremia. The breast milk sodium content was found to be 90 mEq/L. Breastfeeding was stopped, and the baby's hypernatremia was slowly corrected with intravenous (IV) hydration until her sodium level, electrolytes, and renal function tests returned to normal. The infant showed excellent weight gain before discharge.

## 3. Discussion

The typical presentation of neonatal hypernatremic dehydration is between 3 and 21 days of life. Parents may fail to identify their infants' illnesses and be falsely reassured by their apparent well-being [6]. Clinical examination at presentation can vary widely. Some present with lethargy and otherwise normal physical exam, while others may be tonic, active, and hungry but clinically dehydrated, as in our patient.

The sodium content of breast milk varies during the postpartum period, which is highest immediately after delivery (64.8 ± 4.4 mEq/L in term mothers) and declines precipitously by day 3 (21.4 ± 2.3 mEq/L). Then, it keeps on decreasing gradually to reach a mean of 6.9 ± 0.2 mEq/L at 2 weeks postpartum [7]. The sodium breast milk content in our case was 90 mEq/L at 2 weeks postpartum, which is 13-fold higher than normal. Our neonate had no obvious risk for hypernatremia, indicating that breast milk was the source of the high sodium load. Mujawar and Jaiswal [8] reported 8 term neonates who presented with hypernatremic dehydration with elevated breast milk sodium content. The average sodium breast milk content in their study was 40 mEq/L with an average serum sodium level of 164 mEq/L. One baby had both the highest breast milk sodium and serum sodium levels of 88 mEq/L and 186 mEq/L, respectively, both of which are lower than those of our patient. Only one of the 8 neonates presented with excessive weight loss of 30%, as in our patient. None of the neonates was reported to have associated renal failure. Chilton [9] reported a newborn who presented at 7 days of life with lethargy, weight loss of 24%, a serum sodium level of 182 mEq/L, urea of 410 mg/dL, creatinine of 2.4 mg/dL, and breast milk sodium content of 78 mEq/L. Serum electrolytes and renal function tests returned to the baseline with adequate hydration.

One of the most difficult concerns was whether the mother should resume breastfeeding or not. The risks and benefits of breastfeeding, once a high sodium level in breast milk is detected, have not been addressed in the literature. In some reports, breastfeeding was not withheld [8]. However, in our unfortunate situation, our patient had severe electrolyte disturbances with marked acute renal failure, placing her at high risk if she resumed breast milk with such a high sodium content. The decision was discussed with parents to withhold breastfeeding, especially since the follow-up sodium breast milk content after normalization of laboratory values was 80 mEq/L (at 3 weeks of life), which was still significantly elevated.

Correction of hypernatremia should be carried out cautiously since rapid correction is associated with high risk of osmotic change in the brain, with the potential to exacerbate brain edema. High serum sodium leads to the efflux of fluid from the intracellular to the extracellular compartment, with subsequent cerebral cell shrinkage. In return, idiogenic osmoles are created intracellularly to restore brain volume. Therefore, rapid correction of hypernatremia can lead to exacerbation of brain edema by increasing fluid shift intracellularly [10]. For better neonatal outcomes, serum sodium levels should not be corrected at a rate higher than 0.5 mEq/L/hour. However, there is no established consensus for an adequate hypernatremia correction protocol in neonates [4, 11]. Quantified oral feeds allow slow correction of hypernatremia; however, it was not an option in our case due to severe dehydration and renal failure [4]. We were able to slowly correct hypernatremia in our patient with no complications.

The etiologies of high sodium breast milk are not clearly established in the literature. The mother of our baby was healthy with normal serum electrolytes and no past medical history. Moreover, the reported cases in the literature did not establish a causal relationship between the mother and high breast milk sodium content. Further studies are needed to investigate etiologies of high sodium content in breast milk.

The hypernatremia in our case was probably due to dehydration from an inappropriate breast-feeding technique, which given the severe biochemical disturbances, it was majorly exacerbated by the high sodium content in breast milk.

In an attempt to prevent severe consequences associated with neonatal hypernatremic dehydration, it is important to provide proper education on warning signs while ensuring an early postpartum visit to check for signs of dehydration or weight loss. The American Academy of Pediatrics recommends that babies discharged before 48 hours of life should be examined by a healthcare provider within 48 hours of life to weigh the infant, check for hydration status, and ensure good breastfeeding techniques [12]. It would also be important to keep in mind the possibility of high sodium content in breast milk in severe and unexplainable situations.

## 4. Conclusion

Neonatal hypernatremia is a very serious and often fatal condition in exclusively breastfed infants due to inadequate breast milk secretion and feeding difficulties. Monitoring the mother and baby in the early neonatal period for the successful establishment of feeding techniques is of high importance. Neonatal hypernatremia secondary to high sodium content in breast milk is now an established entity and should always be suspected in a healthy baby who presents with weight loss and suspected hypernatremia. Prospective studies are needed to evaluate the need to stop or keep breastfeeding, weighing the risks and benefits and to determine the underlying etiologies of high sodium content in breast milk.

## Data Availability

The data used to support the findings of this study are included within the article.
